# *dms-view*: Interactive visualization tool for deep mutational scanning data

**DOI:** 10.21105/joss.02353

**Published:** 2020-08-17

**Authors:** Sarah K. Hilton, John Huddleston, Allison Black, Khrystyna North, Adam S. Dingens, Trevor Bedford, Jesse D. Bloom

**Affiliations:** 1 Division of Basic Sciences and Computational Biology Program, Fred Hutchinson Cancer Research Center, Seattle, WA, USA; 2 Department of Genome Sciences, University of Washington, Seattle, WA, USA; 3 Vaccine and Infectious Disease Division, Fred Hutchinson Cancer Research Center, Seattle, WA, USA; 4 Molecular and Cell Biology, University of Washington, Seattle, WA, USA; 5 Department of Epidemiology, University of Washington, Seattle, WA, USA; 6 Howard Hughes Medical Institute, Seattle, WA, USA

## Summary and Purpose

The high-throughput technique of deep mutational scanning (DMS) has recently made it possible to experimentally measure the effects of all amino-acid mutations to a protein (Fowler & Fields, 2014, Figure 1). Over the past five years, this technique has been used to study dozens of different proteins (Esposito et al., 2019) and answer a variety of research questions. For example, DMS has been used for protein engineering (Wrenbeck, Faber, & Whitehead, 2017), understanding the human immune response to viruses (Lee et al., 2019), and interpreting human variation in a clinical setting (Gelman et al., 2019; Starita et al., 2017). Accompanying this proliferation of DMS studies has been the development of software tools (Bloom, 2015; Rubin et al., 2017) and databases (Esposito et al., 2019) for data analysis and sharing. However, for many purposes it is important to integrate and visualize the DMS data in the context of other information, such as the 3-D protein structure or natural sequence-variation data. Currently, this visualization requires the use of multiple different tools including custom scripts, static visualization tools like MaveVis (Esposito et al., 2019; Weile, 2019), or protein structure software such as PyMol (Schrödinger, LLC, 2015). No existing tools provide linked views of the protein structure and DMS data in a single interface to facilitate dynamic data exploration and sharing.

Here we describe *dms-view* (https://dms-view.github.io/), a flexible, web-based, interactive visualization tool for DMS data. *dms-view* is written in JavaScript and D3, and links site-level and mutation-level DMS data to a 3-D protein structure. The user can interactively select sites of interest to examine the DMS measurements in the context of the protein structure. *dms-view* tracks the input data and user selections in the URL, making it possible to save specific views of interactively generated visualizations to share with collaborators or to support a published study. Importantly, *dms-view* takes a flexible input data file so users can easily visualize their own DMS data in the context of protein structures of their choosing, and also incorporate additional information such amino-acid frequencies in natural alignments.

**Figure 2:**
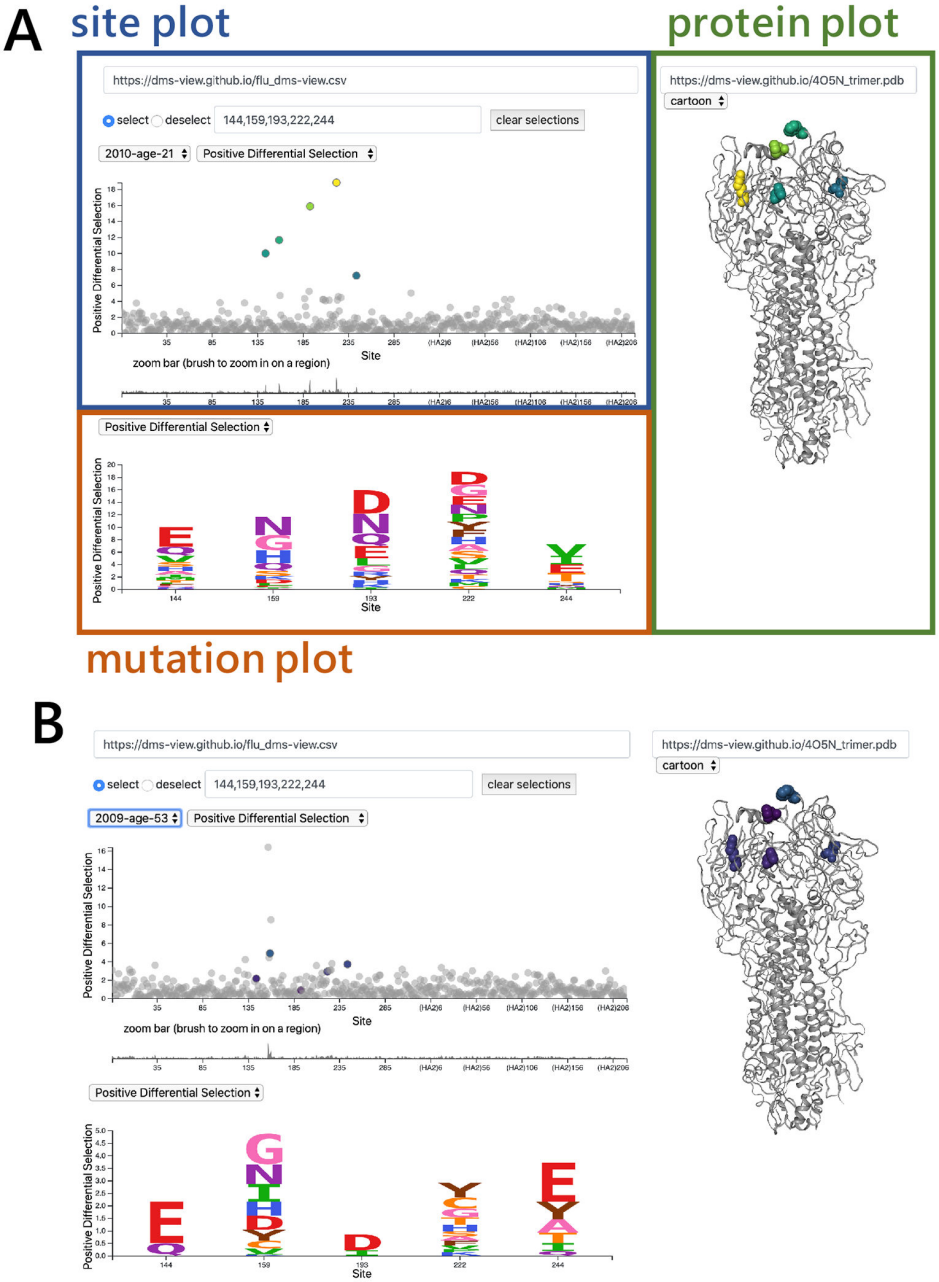
Using *dms-view* to analyze DMS data. For further exploration, please visit https://dms-view.github.io. **(A)** The *dms-view* data section has three panels: the site plot, the mutation plot, and the protein structure plot. The interactive features for selecting sites and navigating are in the site plot panel. Here we show the five sites most highly targeted by human serum “2010-Age-21” from the study by Lee et al. (2019). All five sites fall in the “globular head” of influenza virus HA. **(B)** The same five sites as in panel **A** but now plotted with the data from a different human serum, “2009-age-53”. Using *dms-view* to compare, we see that different sites on HA are targeted by different sera.

Users can access *dms-view* at https://dms-view.github.io. The tool consists of a data section at the top and a description section at the bottom. The data section displays the user-specified data in three panels: the site-plot panel, the mutation-plot panel, and the protein-structure panel (Figure 2A). When sites are selected in the site-plot panel, the individual mutation values are shown in the mutation-plot panel and highlighted on the protein structure. The user can toggle between different conditions, site- and mutation-level metrics, all of which are defined in the user-generated input file. The description section is at the bottom of the page, and allows the user to add arbitrary notes that explain the experimental setup, acknowledge data sources, or provide other relevant information. Note that dms-view is designed to visualize the effects of *single* mutations, not combinations of mutations.

Please visit the documentation at https://dms-view.github.io/docs to learn more about how to use the tool, how to upload a new dataset, or view case studies.

## Example

### Mapping influenza A virus escape from human sera

Using a DMS approach, Lee et al. (2019) measured how all amino-acid mutations to the influenza virus surface-protein hemagglutinin (HA) affected viral neutralization by human sera. For more information on the experimental setup, see the paper (Lee et al., 2019) or the GitHub repo.

We visualized the Lee et al. (2019) serum mapping data using *dms-view*. To explore this dataset, please visit https://dms-view.github.io. In the *dms-view* visualization of these data, the conditions are the different human sera used for the selections. The site- and mutation-level metrics are different summary statistics measuring the extent that mutations escape from immune pressure.

Lee and colleagues asked two questions in their paper which can be easily explored using *dms-view*.

1. *Are the same sites selected by sera from different people?* To explore this question, we compared the site-level and mutation-level metric values for a specific set of sites between different conditions.
2. *Where on the protein structure are the highly selected sites located?* To explore this question, we selected specific sites of interest to be visualized on the 3-D protein structure.

### Comparing site-level and mutation-level metric values for specific sites between conditions

To address whether or not the same sites are selected by different human sera using *dms-view*, we highlighted the most highly targeted sites for the human sera condition “Age 21 2010” in Figure 2A (144, 159, 193, 222, and 244). We then used the condition dropdown menu to toggle to the other sera. The highlighted sites remain highlighted after the condition is changed so we can easily see if the same sites are targeted in other conditions.

In Figure 2B, we can see that there is no overlap of the sites selected by the human sera “2010-age-21” and the human sera “2009-age-53”. These data are the default data for *dms-view*, so to explore this question in more detail please see https://dms-view.github.io.

### View sites on the protein structure

To address where on the protein structure the targeted sites are located, we selected the most highly targeted sites (144, 159, 193, and 222) for the human sera condition “Age 21 2010” to highlight them on the protein structure.

In Figure 2A, we can see that these sites cluster on the “head” of HA, which is known to be a common target of the human immune system (Chambers, Parkhouse, Ross, Alby, & Hensley (2015)).

## Code Availability

- dms-view is available at https://dms-view.github.io.
- Source code is available at https://github.com/dms-view/dms-view.github.io.
- Documentation (https://dms-view.github.io/docs) and case studies (https://dms-view.github.io/docs/casestudies/) are also available.

## Figures and Tables

**Figure 1:**
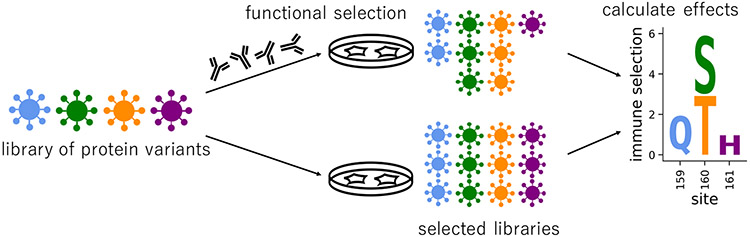
Example deep mutational scanning workflow, modified from Lee et al. (2019). The goal of this experiment is to quantify the how mutations affect a virus’s ability to escape an antibody. The viral variant library contains all single amino-acid changes away from wildtype. The viral library is passaged in cell culture, with and without antibodies, to select for functional variants. Mutational effects are calculated based on deep sequencing of the pre-selected and post-selected libraries.
